# Adherence, virological outcome, and drug resistance in Chinese HIV patients receiving first-line antiretroviral therapy from 2011 to 2015

**DOI:** 10.1097/MD.0000000000013555

**Published:** 2018-12-14

**Authors:** Pengtao Liu, Lingjie Liao, Wei Xu, Jing Yan, Zhongbao Zuo, Xuebing Leng, Jing Wang, Wei Kan, Yinghui You, Hui Xing, Yuhua Ruan, Yiming Shao

**Affiliations:** a Weifang Medical University, Weifang, Shandong Province; b State Key Laboratory for Infectious Disease Prevention and Control, National Center for AIDS/STD Control and Prevention (NCAIDS), Chinese Center for Disease Control and Prevention (China CDC), Collaborative Innovation Center for Diagnosis and Treatment of Infectious Diseases, Beijing; c Guangxi Center for Disease Control and Prevention, Nanning, P. R. China.

**Keywords:** adverse drug reaction, china, drug resistance, antiretroviral therapy, HIV, virological outcome

## Abstract

Stavudine (D4T), zidovudine (AZT), and tenofovir (TDF) along with lamivudine (3TC) are the most widely used HIV treatment regimens in China. China's National Free Antiretroviral Treatment Programme (NFATP) has replaced D4T with AZT or TDF in the standard first-line regimens since 2010. Few studies have evaluated the adherence, virological outcome, and drug resistance in HIV patients receiving first-line antiretroviral therapy (ART) from 2011 to 2015 due to changes in ART regimen.

From 2011 to 2015, 2787 HIV patients were examined, with 364, 1453, and 970 patients having initiated D4T-, AZT-, and TDF-based first-line ART regimens, respectively. The Cochran–Armitage test was used to examine the trends in clinical and virological outcomes during 2011 to 2015. Logistic regression was used to examine the effects of different regimens after 9 to 24 months of ART.

From 2011 to 2014–2015, adverse drug reactions decreased from 18.9% to 6.7%, missed doses decreased from 9.9% to 4.6%, virological failure decreased from 16.2% to 6.4%, and drug resistance rates also significantly decreased from 5.4% to 1.1%. These successes were strongly associated with the standardized use of TDF- or AZT-based regimens in place of the D4T-based regimen. Poor adherence decreased from 11.3% in patients who initiated D4T-based regimens to 4.9% in those who initiated TDF-based regimens, adverse drug reactions decreased from 32.4% to 6.7%, virological failure reduced from 18.7% to 8.6%, and drug resistance reduced from 5.8% to 2.9%. Compared with patients who initiated AZT-based regimens, patients who initiated TDF-based regiments showed significant reductions in adherence issues, adverse drug reactions, virological outcomes, and drug resistance. Significant differences were also observed between those who initiated D4T- and AZT-based regimens.

The good control of HIV replication and drug resistance was attributed to the success of China's NFATP from 2011 to 2015. This study provided real world evidence for further scaling up ART and minimizing the emergence of drug resistance in the “Three 90” era.

## 1 Introduction

Although stavudine (D4T) and zidovudine (AZT) are not recommended as first-line therapy in well-resourced settings, these thymidine analogues, along with lamivudine (3TC), have formed the most widely used combinations in LMICs.^[1,2]^ In many of Asia and other resource-limited settings, the most common first-line regimens for HIV-infected patients still contain either D4T or AZT.^[1,3,4]^ But over the last decade, antiretroviral therapy (ART) has gradually improved survival rate and life quality of patients with HIV in low- and middle-income countries (LMICs).^[5–9]^ If ART regimens are not effectively delivered, HIV drug resistance (HIVDR) could become widespread, leading to an increase in therapeutic failures, transmission of drug-resistant viruses, an effectiveness decrease in treatment program, and a decrease in overall patient survival. Therefore, important questions still focus on best practice of ART and how to successfully promote on-going treatment in LMICs.^[10]^

As of December 31, 2015, 760,305 people were reported HIV-infected in China, 471,140 of whom had been treated through the China National Free Antiretroviral Treatment Programme (NFATP).^[11]^ Long-term antiviral therapy is necessary for most patients; however, incomplete viral suppression and emergence of drug resistance have been major problems to be concerned.^[12,13]^ According to the World Health Organization (WHO) ART guidelines, tenofovir (TDF) is the preferred first-line regimen for adults and adolescents.^[14]^ Accordingly, in 2011, the NFATP expanded the use of TDF, and 6974 patients received TDF-based regimens as first-line therapy this year; this number increased to 116,726 in 2014, accounting for about 40.1% of all patients in NFATP. The use of different ART regimens in China permitted us to conduct a comparison of TDF-based regimens versus other ART regimens in antiretroviral naive HIV-infected individuals in China.

Due to the changes and the rapid scale-up of ART in China, the aim of this study was to evaluate the impact and consequences of NFATP guideline changes. Specifically, we investigated: the efficacy of D4T and AZT compared to TDF for HIV patients receiving first-line ART in clinical practice.

## 2 Methods

### 2.1 Ethical approvals

All subjects provided written informed consent to participate in this study. The institutional review board of the National Center for AIDS/STD Control and Prevention (NCAIDS), Chinese Center for Disease Control and Prevention (China CDC) approved this study (ethical approval number: X140617334).

### 2.2 Study design and study population

**Figure 1:**
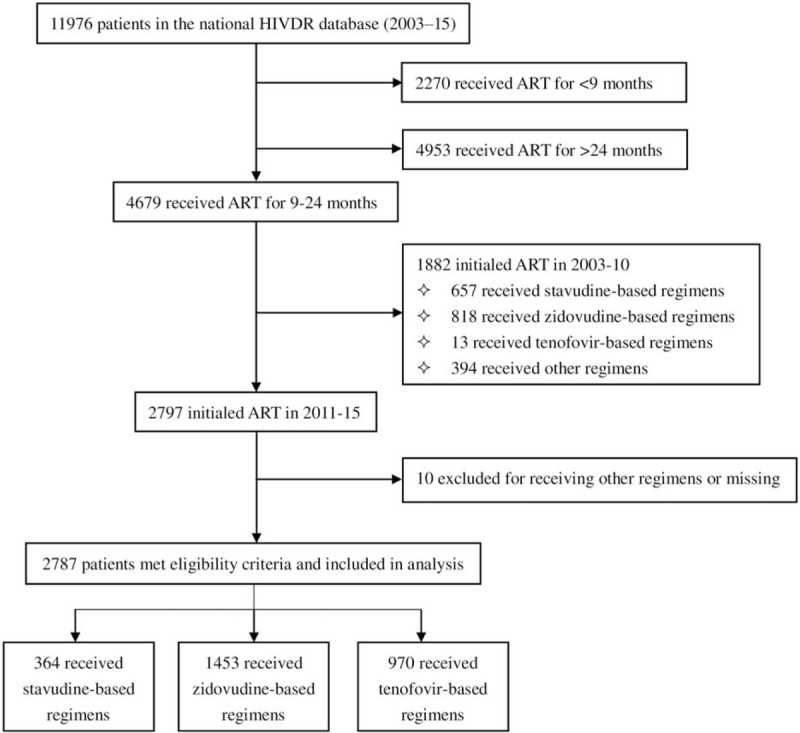
In total, 2787 patients were included in our study. Patients were stratified based on first-line regimen: 364 patients initiated D4T-based ART [D4T/3TC/efavirenz (EFV) or nevirapine (NVP)], 1453 patients initiated AZT-based ART (AZT/3TC/EFV or NVP) and 970 patients initiated TDF-based ART (TDF/3TC/EFV or NVP) from 2011 to 2015.

Data were extracted from the Chinese National HIVDR Surveillance database as previously described.^[1]^ The surveillance protocol was adopted from the WHO recommended surveillance of HIVDR in adults receiving ART.^[15]^ Of 11,976 participants from the national HIVDR database, a total of 4679 patients met our eligibility criteria of having received first-line ART for 9 to 24 months. Of these, 1882 patients initiated ART before 2011 and 2010 were excluded for missing values (Fig. 1). The inclusion criteria for this study selected to use about study population were as follows: ≥18 years old in the survey; 9 to 24 months of ART; treatments in the NFATP were D4T-, AZT-, or TDF-based first-line ART regimens; having blood specimens collected for VL testing; and consent and willingness to participate in the previously conducted surveillance study. Patients were excluded if they were not initially treated through the NFATP.

### 2.3 Laboratory tests

All subjects provided blood specimens for testing of CD4 cell counts, HIV-1 RNA viral load, and HIV drug resistance mutations at baseline and 12-month follow-up. Blood samples were collected by the local CDC. CD4 cell counts at enrolment were determined with flow cytometry (Fascount, Becton Dickinson, San Jose, CA). Plasma HIV-1 RNA levels were determined using the HIV Generic Viral Load assay (Biocentric, Bandol, France) as previously described.^[1,16,17]^ Successful viral suppression was defined as HIV-1 RNA viral load <1000 copies/mL. Genotypic and drug resistance tests were performed among viremic patients with virological failure (defined as a HIV-1 RNA viral load ≥1000 copies/ml, according to the WHO 2010 recommendations) after 6 months of treatment since the start of the study as previously described.^[1,16,17]^ Drug resistance was determined according to the HIV Drug Resistance Database Program version 8.4 (https://hivdb.stanford.edu/, updated on 2017-06-16). All drug resistance mutations that conferred low, intermediate, or high resistance were included.^[18]^

### 2.4 Statistical analysis

We performed a retrospective analysis on data from national HIV drug resistance surveys. The primary outcome variables were adverse drug reaction (defined as any untoward medical occurrence, irrespective of its suspected relationship to the study medications per International Conference of Harmonization (ICH) guidelines.), adherence, virological outcomes, and drug resistance. The data were analyzed using unadjusted odds ratios with a test for significance according to *χ*^2^ tests and Fisher exact test. Cochran–Armitaget test was used to examine the trends in clinical and virological outcomes during 2011 to 2015. Multivariable logistic regression was performed to analyze adjusted odds. *P* < .05 was considered statistically significant and all reported values were 2-sided. Data analyses were performed using SAS software (SAS 9.4 for Windows, Weifang, Shandong Province, China; SAS Institute Inc., NC).

## 3 Results

### 3.1 Basic characteristics of the participants

Table 1 shows the basic characteristics of the study population. In summary, 78.3% were aged <40 years; 78.3% belonged to the Han ethnic group; 74.1% were men; 7.5% had a primary school education (1–6 years of education) or less; 44.6% were married; 46.6% were farmers; and 77.4% were infected through sexual intercourse. The proportions of patients with baseline (pre-ART) CD4 counts of ≥350, 200 to 349, and 50 to 199 cells/mm^3^ were 19.4%, 37.7%, and 28.3%, respectively (Table 1).

**Table 1:**
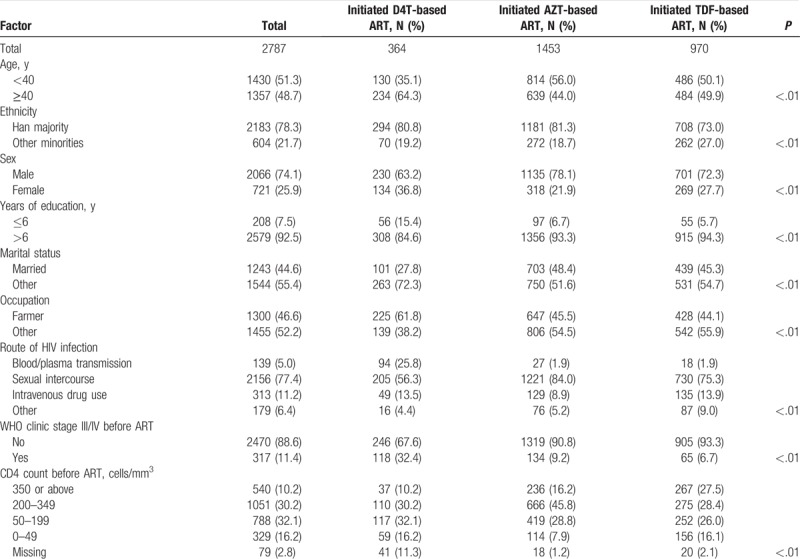
Basic characteristics of participants who initiated first-line ART from 2011 to 2015, stratified by initial antiretroviral therapy regimens.

### 3.2 Changes of treatment outcomes in HIV patients initiating first-line ART from 2011 to 2015

The Cochran–Armitage test was used to examine the trends in adverse drug reaction, missed dose in the past month, virological failure (HIV RNA ≥1000 copies/mL), drug resistance, and initial ART regimens in HIV patients who initiated first-line ART during 2011, 2012 to 2013, and 2014 to 2015.

The prevalence of adverse drug reactions in the past month decreased from 18.9% to 6.7% in 2011 to 2014–2015 (*P* < .01). Missed doses in the past month, virological failure (HIV RNA ≥1000 copies/mL), and drug resistance decreased from 9.9% to 4.6% (*P* < .01), 16.2% to 6.4% (*P* < .01), 5.4% to 1.1% (*P* < .01), respectively, from 2011 to 2014–2015. Significant reductions in drug resistance of nonnucleoside reverse transcriptase inhibitor (NNRTI) (NVP or EFV), nucleoside reverse transcriptase inhibitor (NRTI), PI, NNRTI, and NRTI were also observed, respectively. It was worth noting that the rates of patients who initiated TDF-based ART were gradually increasing in the last few years (16.0% in 2011, 35.4% in 2012–2013, and 58.1% in 2014–2015, respectively, *P* < .01) (Table 2).

**Table 2:**
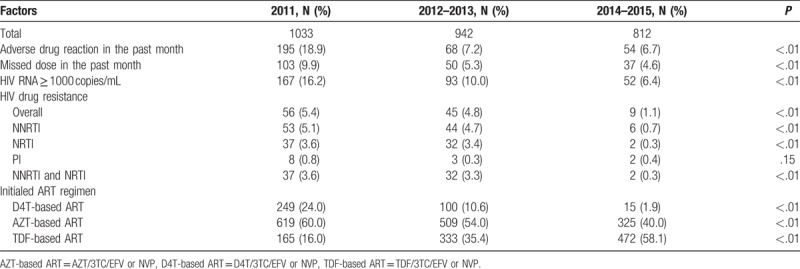
Changes in virological outcomes and drug resistance in patients who initiated first-line antiretroviral therapy from 2011 to 2015.

### 3.3 Comparison of treatment outcomes between HIV patients initiating D4T- and TDF-based first-line ART

After adjustment for age (as categorical, Table 1), ethnicity, education, sex, marital status, occupation, route of HIV transmission, and CD4 count (as categorical, Table 1) before ART, patients who initiated TDF-based ART had 62% lower odds of having missed doses in the past month [adjusted OR (AOR), 0.38; 95% CI, 0.24–0.60], 83% lower odds of adverse drug reactions (AOR, 0.17; 95% CI, 0.12–0.24), 56% lower odds of virological failure (AOR, 0.44; 95% CI, 0.30–0.64), and 52% lower odds of HIVDR (AOR, 0.48; 95% CI, 0.27–0.87). After adjustment for age (as categorical), ethnicity, education, sex, marital status, occupation, route of HIV transmission, CD4 count (as categorical) before ART, and missed doses in the past month, patients who initiated TDF-based regimens had significantly lower odds of resistance to NNRTI (*P* < .01) and NRTI (*P* < .01) class drugs, as well as multidrug resistance (MDR) to NNRTI and NRTI drugs (*P* < .01); however, they had higher odds of resistance to PI class drugs (*P* = .76) (Table 3).

**Table 3:**
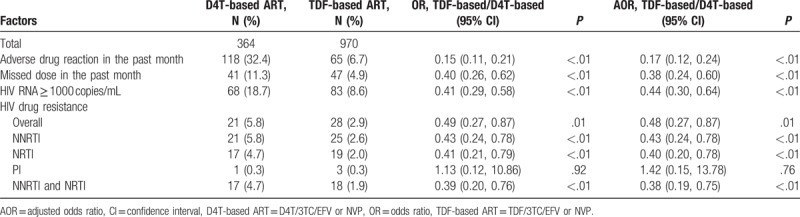
Virological outcomes and drug resistance in patients who initiated stavudine- and tenofovir-based first-line antiretroviral therapy from 2011 to 2015.

### 3.4 Comparison of treatment outcomes between HIV patients initiating D4T- and AZT-based first-line ART

After adjustment for age (as categorical), ethnicity, education, sex, marital status, occupation, route of HIV transmission, and CD4 count (as categorical) before ART, patients who initiated AZT-based ART had 41% lower odds of having missed doses in the past month [adjusted OR (AOR), 0.59; 95% CI, 0.39–0.89], 75% lower odds of adverse drug reactions (AOR, 0.25; 95% CI, 0.19–0.34), and 40% lower odds of virological failure (AOR, 0.60; 95% CI, 0.30–0.64). After adjustment for age (as categorical), ethnicity, education, sex, marital status, occupation, route of HIV transmission, CD4 count (as categorical) before ART, and missed doses in the past month, patients who initiated AZT-based regimens had significantly lower odds of resistance to NRTI (*P* = .04) class drugs, as well as MDR to NNRTI and NRTI drugs (*P* = 0.04); however, they had higher odds of resistance to PI class drugs (*P* = .21) (Table 4).

**Table 4:**
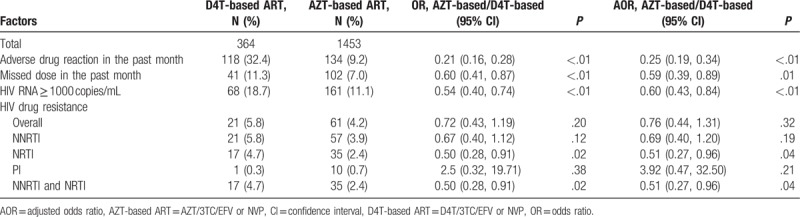
Virological outcomes and drug resistance in patients who initiated stavudine- and zidovudine-based first-line antiretroviral therapy from 2011 to 2015.

### 3.5 Comparison of treatment outcomes between HIV patients initiating AZT- and TDF-based first-line ART

After adjustment for age (as categorical), ethnicity, education, sex, marital status, occupation, route of HIV transmission, and CD4 count (as categorical) before ART, patients who initiated TDF-based ART had 32% lower odds of having missed doses in the past month [adjusted OR (AOR), 0.68; 95% CI, 0.48–0.98], 29% lower odds of adverse drug reactions (AOR, 0.71; 95% CI, 0.52–0.97), 30% lower odds of virological failure (AOR, 0.70; 95% CI, 0.52–0.93), and 39% lower odds of HIVDR (AOR, 0.61; 95% CI, 0.38–0.97). After adjusting for age (as categorical), ethnicity, education, sex, marital status, occupation, route of HIV transmission, CD4 count (as categorical) before ART, and missed doses in the past month, patients who initiated TDF-based regimens had significantly lower odds of resistance to NNRTI (*P* = .03) class drugs (Table 5).

**Table 5:**
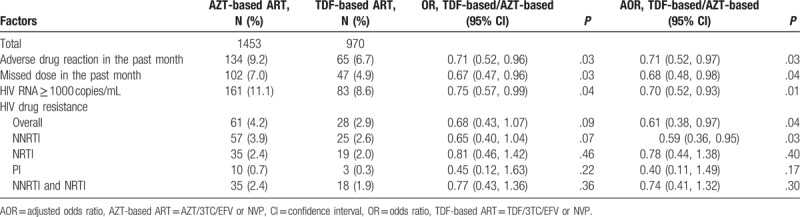
Virological outcomes and drug resistance in patients who initiated zidovudine- and tenofovir-based first-line antiretroviral therapy from 2011 to 2015.

## 4 Discussion

Our results demonstrated significant improvements in adherence, virological outcome, and drug resistance in Chinese HIV patients receiving first-line ART from 2011 to 2015, which persisted after adjusting for other factors. In this large-scale study, significant improvements in adverse drug reaction (18.9–6.6%, *P* < .01), missed dose (9.9–4.6%, *P* < .01), virological failure (16.7–5.6%, *P* < .01), and drug resistance (6.0–1.1%, *P* < .01) were observed from patients receiving ART over the study period. Significant reductions in drug resistance of NNRTI, NRTI, and PI were also observed, respectively. The results were consistent with prior studies suggesting that the reductions in virological failure and HIVDR may be partly attributable to improvements in treatment and care, such as replacement of DDI with 3TC as the first-line treatment in NFATP,^[1]^ changes of social demographic factors among patients,^[16,17,19–21]^ and providing high-quality care to HIV/AIDS patients.^[19]^

The virologic outcomes and HIVDR in this study were better than the outcomes of previous surveillance studies in China.^[1,17,19]^ It is also better than the outcomes in studies of ART from some developing countries worldwide including Angola (16%), Cuba (22%), Papua New Guinea (16%), and South Africa (14%).^[22]^ A recent report from the WHO showed that the prevalence of HIVDR has increased from 11% to 29% since the global rollout of ART in 2001.^[23]^ So to achieve “the last 90” target of the Joint United Nations Program on HIV/AIDS (UNAIDS) (90% of people with HIV infection know they have it, 90% of those infected are receiving ART, and sustained viral suppression is achieved in 90% of those receiving treatment.^[24]^), multifaceted interventions should be warranted.

This study showed that the reductions in virological failure and drug resistance since 2011 were strongly associated with the standardized use of TDF- or AZT-based regimens in place of the D4T-based regimen. This highlighted the right decision for phasing out D4T in favor of TDF for first-line ART in NFATP in the last few years. D4T was initially recommended by the WHO largely due to a lower cost than AZT. However, newer ART agents, including TDF, are effective and safer than older nucleoside reverse-transcriptase inhibitor agents, although they might be associated with nephrotoxicity.^[25]^ The first-line ART regimen in the second edition of the NFATP Guideline in 2008 was revised to consist of TDF/AZT + 3TC + EFV/NVP, and NFATP expanded the use of TDF in 2011 according to the WHO guidelines.^[1,26]^ We assessed the effects of D4T-, AZT-, and TDF-based regimens as first-line therapy in China's NFATP on ART-related adverse drug reactions, adherence, virological outcomes, and HIVDR. Virological failure decreased from 18.7% in patients who initiated D4T-based regimens to 8.6% in those who initiated TDF-based regimens, and drug resistance reduced from 5.8% to 2.9%. The results were in line with previous studies.^[4]^ However, consequences of TDF use remained to be determined in some LMICs, where viral load monitoring was limited.^[27]^ In these countries, HIVDR remained a very serious concern.^[6,28–31]^

Significant differences were also observed between those who initiated AZT- and TDF-based regimens. A study from South Africa demonstrated that TDF-based regimens were associated with fewer adverse drug reactions and lower proportions of loss-from-care compared to AZT-based regimens.^[32]^ This result was consistent with our study that adverse drug reactions and adherence issues at 9 to 24 months of treatment decreased from 9.2% and 7.0% in patients who initiated AZT-based regimens to 6.7% and 4.9% in those who initiated TDF-based regimens, respectively. Improvements in adherence were largely attributable to decreased adverse drug reactions, which were a major concern with AZT/D4T treatment regimens.^[33,34]^

In addition to the role of ARV drugs, age, and sex were other important factors that affected adherence, virological outcomes, and drug resistance according to previous studies.^[17,19]^ We conducted a supplementary analysis to investigate the effects of age and sex in adherence, virological outcomes, and drug resistance among patients who initiated ART in this study. The results showed that patients over 40 years old were less likely to miss dose in the past month (AOR, 0.62; 95% CI, 0.45–0.85). However, the effect of age was not significant in virological outcomes and drug resistance. Female patients were less likely to have virological failure (AOR, 0.72; 95% CI, 0.54–0.98). However, the effect of sex was not significant in adherence (miss dose in the past month) and drug resistance. In order to learn more about the effect of age and sex in ART and develop therapies against treatment failure, further studies will be needed to confirm the effect of age and sex in adherence, virological outcomes, and drug resistance among patients who initiate ART in China.

Our study has several limitations. First, it was limited by its cross-sectional nature; early failure and mortality were not studied. In 2015, 18.9% patients being excluded due to mortality, treatment termination and loss to follow-up likely introduced selection bias; their numbers were probably insufficient to alter the conclusions derived from multivariable modeling. Second, ART regimens prescribed differently to groups of patients with varying baseline characteristics and prognoses might give rise to selection bias. The use of specific drugs/regimens for people with specific characteristics could influence the outcomes of these drugs/regimens. This was supported by the fact that D4T users were, among other characteristics as described in Table 1, older, more often female and less educated compared to the other groups. Third, baseline HIV viral load distribution was not described and included as a variable for adjustment in the multivariate analyses because of missing values. After adjusting for factors measured at the initiation of NFATP, there was an improvement in outcomes of ART. Compared to previous study that most patients receiving TDF or AZT-based regimens achieved good therapeutic effect,^[19,35]^ more factors were considered in this paper and the solution was closer to reality. However, other important confounding factors, such as viral load and the 3rd drugs which would change during the ART, were not included in this study.

In the end, this study confirmed that the success of ART scale-up in resource-limited settings was largely due to the introduction of a public health approach to access treatment advocated by the WHO that emphasized standardized treatment regimens that could be purchased in large quantities and delivered at scale.

## Acknowledgments

The authors would like to thank Yi Feng, Ruihua Kang, Yang Li, Zheng Li, Jing Zhang, Jing Hu, Yang Li, Shuai Zhao, Pi Cao, Cui He, Jenny H. His, Xiaoqin Xu, Yong Liu, Jianmei He, Hua Ling, Ping Ding, Yi Tong, Xiaobai Zou, Quanhua Zhou, Xia Wang, Yanling Ma, Bin Su, Jihua Fu, Jianmei He, Lin Chen, Xiaohong Pan, Yonghui Dong, Wei Liu, Liting Yang, Jingyun Li, Hong Shang, Ping Zhong, Hanping Li, Min Zhang, Yile Xue, Zhe Wang, Bin Su, Wei Liu, Yonghui Dong, Yanling Ma, Huiqin Li, Guangming Qin, Lin Chen, Xiaohong Pan, Xi Chen, Guoping Peng, Jihua Fu, Ray Y. Chen, Laiyi Kang and who all gave valuable comments or collected the data. We thank the patients and doctors throughout China for their consent and support for the study.

## Author contributions

**Conceptualization:** Pengtao Liu, Lingjie Liao, Hui Xing, Yuhua Ruan.

**Data curation:** Lingjie Liao, Wei Xu, Jing Yan, Zhongbao Zuo, Xuebing Leng, Wei Kan, Yinghui You, Hui Xing, Yuhua Ruan.

**Formal analysis:** Pengtao Liu, Xuebing Leng, Wei Kan, Hui Xing, Yuhua Ruan.

**Funding acquisition:** Hui Xing, Yuhua Ruan, Yiming Shao.

**Investigation:** Lingjie Liao, Wei Xu, Jing Yan, Zhongbao Zuo, Xuebing Leng, Jing Wang, Hui Xing, Yuhua Ruan.

**Methodology:** Pengtao Liu, Xuebing Leng, Hui Xing, Yuhua Ruan.

**Project administration:** Pengtao Liu, Hui Xing, Yuhua Ruan, Yiming Shao.

**Resources:** Lingjie Liao, Wei Xu, Hui Xing, Yuhua Ruan.

**Software:** Pengtao Liu.

**Supervision:** Pengtao Liu, Lingjie Liao, Wei Kan.

**Validation:** Lingjie Liao, Wei Xu, Jing Yan, Xuebing Leng.

**Visualization:** Pengtao Liu, Lingjie Liao, Jing Yan, Xuebing Leng.

**Writing – original draft:** Pengtao Liu, Yinghui You.

**Writing – review & editing:** Pengtao Liu, Yinghui You, Yuhua Ruan.
